# FGFR1-Induced Epithelial to Mesenchymal Transition through MAPK/PLCγ/COX-2-Mediated Mechanisms

**DOI:** 10.1371/journal.pone.0038972

**Published:** 2012-06-12

**Authors:** Darren C. Tomlinson, Euan W. Baxter, Paul M. Loadman, Mark A. Hull, Margaret A. Knowles

**Affiliations:** 1 Section of Experimental Oncology, Leeds Institute of Molecular Medicine, St. James’s University Hospital, Leeds, United Kingdom; 2 Instititue of Cancer Therapeutics, University of Bradford, Richmond Road, Bradford, United Kingdom; 3 Section of Molecular Gastroenterology, Leeds Institute of Molecular Medicine, St. James’s University Hospital, Leeds, United Kingdom; University of Nebraska Medical Center, United States of America

## Abstract

Tumour invasion and metastasis is the most common cause of death from cancer. For epithelial cells to invade surrounding tissues and metastasise, an epithelial-mesenchymal transition (EMT) is required. We have demonstrated that FGFR1 expression is increased in bladder cancer and that activation of FGFR1 induces an EMT in urothelial carcinoma (UC) cell lines. Here, we created an in vitro FGFR1-inducible model of EMT, and used this model to identify regulators of urothelial EMT. FGFR1 activation promoted EMT over a period of 72 hours. Initially a rapid increase in actin stress fibres occurred, followed by an increase in cell size, altered morphology and increased migration and invasion. By using site-directed mutagenesis and small molecule inhibitors we demonstrated that combined activation of the mitogen activated protein kinase (MAPK) and phospholipase C gamma (PLCγ) pathways regulated this EMT. Actin stress fibre formation was regulated by PLCγ activation, and was also important for the increase in cell size, migration and altered morphology. MAPK activation regulated migration and E-cadherin expression, indicating that combined activation of PLCγand MAPK is required for a full EMT. We used expression microarrays to assess changes in gene expression downstream of these signalling cascades. COX-2 was transcriptionally upregulated by FGFR1 and caused increased intracellular prostaglandin E_2_ levels, which promoted migration. In conclusion, we have demonstrated that FGFR1 activation in UC cells lines promotes EMT via coordinated activation of multiple signalling pathways and by promoting activation of prostaglandin synthesis.

## Introduction

Epithelial to mesenchymal transition (EMT) is a process that was observed initially in embryonic development but more recently has been implicated as a mechanism for cancer metastasis [1], [2]. Although tumour invasion and metastasis is the major cause of death in cancer patients, the biological mechanisms of metastasis remain incompletely understood. The majority of adult solid tumours are derived from an epithelial lineage. Epithelial cells form layers of cells that are closely adjoined by specialised membrane structures and such cells are generally non-motile under normal conditions. For epithelial cancer cells to invade into surrounding tissues and establish secondary tumours at distant sites they must lose cell-cell adhesions and polarity and increase their motility. Understanding the complex mechanisms that drive these changes in EMT is key to developing therapeutic strategies to both prevent and treat metastasis.

Many advances in understanding the mechanisms that promote EMT, including the identification of transcription factors and other proteins that play key roles in these processes [3], have come from studies of cell culture models [4], . In such systems, a variety of extracellular signals can activate an EMT: these include components of the extracellular matrix, soluble factors such as members of the fibroblast growth factor (FGF) and transforming growth factor β (TGFβ) families, epidermal growth factor, hepatocyte growth factor and others [2]. Interestingly, some factors that under normal physiological conditions regulate proliferation or differentiation rather than EMT, are essential for inducing EMT-specific events in pre-malignant epithelial cells [9]. Pre-malignant cells frequently gain their ability to proliferate and clonally expand due to constitutive activation of receptor tyrosine kinases and downstream effectors such as RAS. Several studies have demonstrated cooperation between growth factors and RAS signalling in the induction of EMT [10], [11], [12] suggesting that coordinated activation of multiple pathways is essential for EMT to occur.

Bladder cancers frequently show increased signalling via FGF receptors (FGFRs) [13],[14],[15]. These tumours comprise at least two major disease entities, with distinct molecular profiles [16], [17]. Activating mutations in *FGFR3* are found at high frequency in low-grade non-invasive (stage Ta) urothelial carcinoma (UC) [18] and several studies have highlighted activated FGFR3 as a potential therapeutic target in this subgroup [19], [20], [21]. As many muscle-invasive (stage ≥T2) UC show upregulation of non-mutant FGFR3 [14], this may also be a valid therapeutic target in these poor prognosis cancers.

A high proportion of UC of all grades and stages also show upregulated expression of FGFR1 [21]. In normal urothelial cells, we have shown that FGFR1 signalling stimulates proliferation and increases cell survival but does not induce invasion or changes in cell motility. In prostate cancer, activation of FGFR1 can mediate EMT (reviewed in [22]), raising the question whether FGFR1 signalling may play a different role in invasive compared to non-invasive bladder tumours. This prompted us to investigate the ability of FGFR1 activation to induce EMT in UC-derived cell lines. Here we show that ligand-induced activation of ectopically expressed FGFR1 can promote an EMT-like phenotype, with a decrease in E-cadherin expression, morphological changes and increased migration and invasion. Using site-directed mutagenesis and small molecule inhibitors we have identified signalling pathways activated by FGFR1 that contribute to EMT, including the MAPK, PLCγ and COX-2 signalling pathways. Our findings suggest potential therapeutic approaches that may be applicable in the prevention and/or treatment of UC metastasis.

## Materials and Methods

### Ethics Statement

The cell line LUCC3 was established in our laboratory from a primary tumour sample obtained with written patient consent and approval from the Leeds-East Research Ethics Committee.

### Cell Culture and Production of Retroviruses and Transductions

Cell lines 96-1, 94-10, 97-7 [23], [24], J82 [25], VMCUB3 [26], LUCC3 (Pitt *et al.* unpublished) and telomerase-immortalized normal human urothelial cells (NHUC) [27] were used. All lines have been authenticated in our laboratory by extensive genomic analysis (microsatellite typing, conventional karyotypic analysis, MFISH, array-based copy number analysis and mutation analysis). Cells were grown in standard growth media at 37°C in 5% CO_2_. NHUC were maintained as described [27]. FGFR1 (NM_023110) and FGFR1 Y766F were cloned as described [21]. FGFR1 and FGFR1 Y766F constructs were transfected into Phoenix A packaging cells (ATCC), using siPORT™ XP-1 transfection agent (Ambion). After 48 hours, medium was harvested, 0.4 µm filtered and mixed in equal amounts with fresh medium containing 8 µg/ml of polybrene (Sigma). Cells were incubated with retroviral supernatants for 8 hours. Forty-eight hours after transduction, cells were transferred into selection medium containing hygromycin.

### Quantitative Real-time Reverse Transcriptase-PCR for FGFRs

Total RNA was extracted from cell lines. RNA was extracted using Qiagen RNeasy Mini Kit (Qiagen) and 1 µg was reverse transcribed in the presence or absence of reverse transcriptase (Invitrogen) according to the manufacturer’s instructions. Real-time RT-PCR analysis was performed using E-cadherin and COX-2 Taqman probes and normalised to SDHA as an internal control (Applied Biosystems).

### Western Blotting, Cell Staining and PGE2 Analysis

Cells were lysed in Triton buffer [1% Triton-X 100, 1 mM EDTA, and protease inhibitor cocktail (Sigma) in PBS] and lysates cleared by centrifugation at 10000 rpm at 4°C. The protein concentrations were determined using the BCA (bicinchonic acid) assay (Pierce). Antibodies used for western blotting were anti-phospho-ERK (sc-7383, Santa Cruz), ERK (sc-154, Santa Cruz), phospho-PLCγ (2821, Cell Signaling), PLCγ Cell Signaling), phospho-STAT3 (9135, Cell Signaling), STAT3, phospho-p38 (9221, Cell Signaling), p38 (9217, Cell Signaling), phospho-ATF (9221, Cell Signaling), phospho-MEK1/2 (9121, Cell Signaling), FGFR1 (SC-121, Santa Cruz), E-cadherin (ab1416, Abcam), plakoglobin (p8087, Sigma) and tubulin (MCA77G, Serotec). The Human Phospho-Kinase Antibody Array (R&D Systems) was performed as described by the manufacturer. Briefly, the array was blocked then incubated with 200 µg of protein extracted from treated cells, prior to incubation with Detection Antibody Cocktail. The Arrays were imaged and quantified using Quantity One Software (BioRad).

For cell staining, cells were fixed on glass slides with 4% paraformaldehyde (PFA) for 15 min, washed in PBS and permeabilized with 0.25% triton/PBS for 5 min. Cells were washed in PBS and incubated with Phalloidin-488 (Molecular Probes) and DAPI.

For prostaglandin E_2_ (PGE_2_) analysis, cells were cultured with FGF2 or arachidonic acid (Sigma) for 4 hours. Cells lysates were made by repeated freeze thawing cycles. PGE_2_ was analysed in media and lysates using the Prostaglandin E2 EIA Kit (Cayman Chemical).

### Phenotypic Assays

For transwell assays 5×10^5^ cells were plated per transwell in a 24 well dish in duplicate per experiment. Each experiment was repeated three times. 5 hr after plating, media was replaced with serum-free media with the appropriate culture conditions and left for 24–36 hr (heparin, 10 µg/ml; FGF2, 10 ng/ml; U0126, 10 µM; BAPTA-AM, 10 µM; BIRB, 1 µM; NS-398, 20 µM; AH-23848, 20 µM). Cultures were pre-treated for one hour (on both sides of the chamber) prior to the addition of FGF2 to the lower chamber. The cells that remained on the upper side of the transwell were scraped off and the cells that migrated across the transwell were fixed in methanol/acetone for 5 min. Cells were stained with DAPI and the transwell imaged using Volocity software. DAPI stain was quantified using the Volocity software. For the Matrigel™ assays, a layer of Matrigel™ (Invitrogen), mixed with an equal amount of media containing heparin or heparin and FGF2, was placed in the transwell. 1×10^5^ cells were plated on the Matrigel™, media was replaced after 6 hr and the cells were incubated for 96 h. The cells were fixed in 4% PFA for 30 min, permeabilized with 0.25% triton/PBS and incubated with Phalloidin-Alexa 488. Multiple points on each well were imaged using a confocal microscope. For scratch assays cells were plated at high density and cultured until confluence. Cells were scratched with a pipette tip in two directions to enable imaging, washed twice in serum free media, and the appropriate supplements added. Cells were imaged after 48 hours. Cell size was measured using the Guava EasyCyte™ System (Guava Technologies), according to the manufacturer’s instructions.

## Results

### FGFR1 Activation Induces EMT in UC Cell Lines

**Figure 1:**
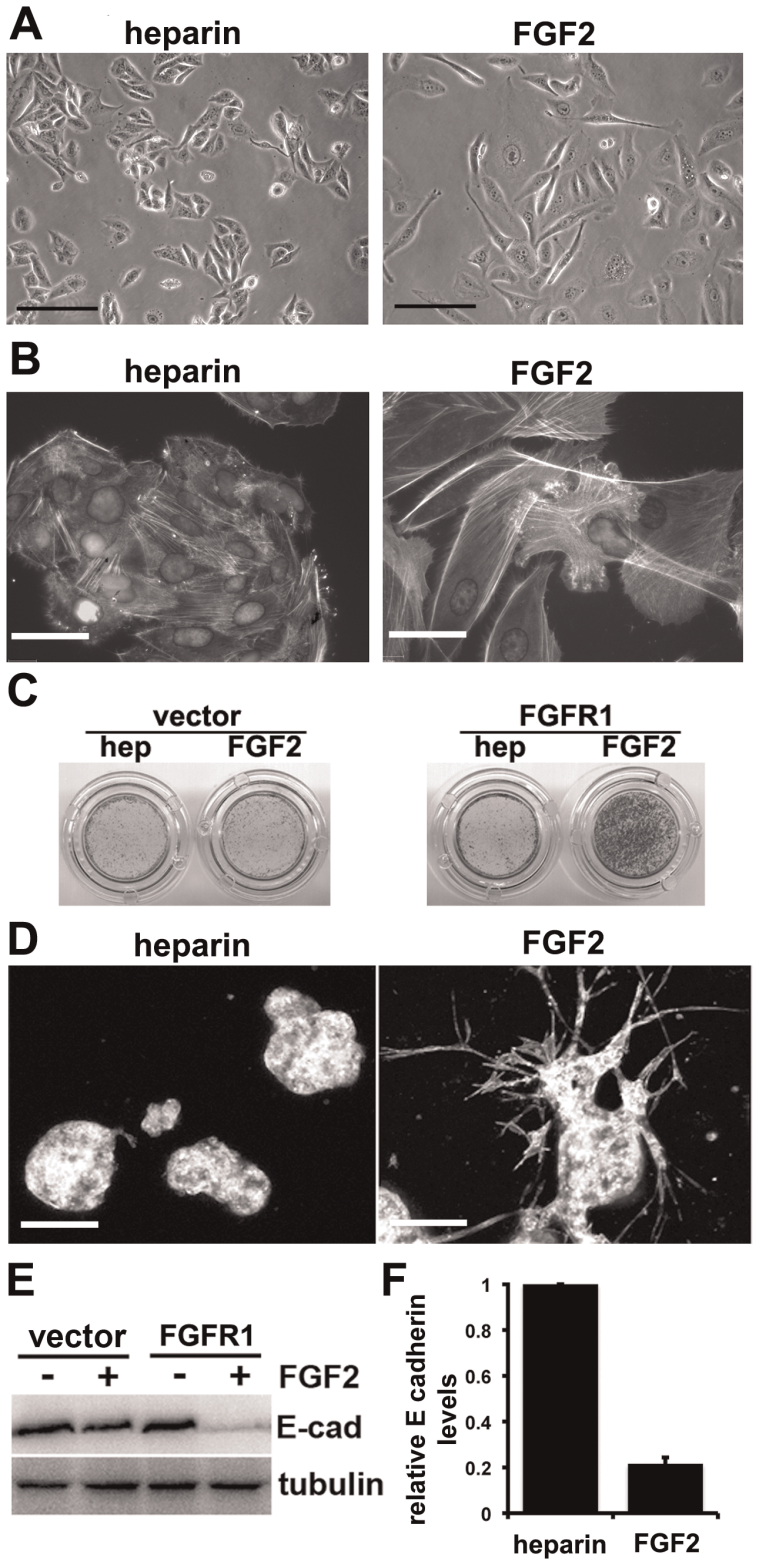
FGFR1 activation promotes EMT in 94-10 cells. A. 94-10-FR1 cells were cultured with heparin or heparin and FGF2 for 72 h. Images were taken at 72 h (bars = 100 µm). B. At 72 h 94-10-FR1 cells cultured with heparin or heparin and FGF2 were fixed and stained with DAPI and Phalloidin-Alexa 488 (bars = 30 µm). Images represent a merged picture of DAPI and Phalloidin-Alexa 488 staining. C. Transwell assays were used to assess changes in migration in 94-10-FR1 and control cells. Cells which had migrated (cells on the lower part of the transwell) were stained at 36 hr with haematoxylin. D. Invasion assays were performed on 94-10-FR1 cells seeded onto a layer of 50% matrigel containing heparin or heparin and FGF2. At 96 h they were fixed, stained with Phalloidin and imaged using confocal microscopy (bars = 0.5 mm). E. Western blots for E-cadherin and tubulin (loading control) on 94-10-FR1 and 94-10 vector control cells cultured with heparin or heparin and FGF2 for 72 h. F. Real time RT-PCR for E-cadherin on 94-10-FR1 cells cultured with heparin or heparin and FGF2 for 72h. SDHA was used an internal control.

UC cell lines express several FGFR family members (our unpublished data). Thus, stimulation with FGFs may activate more than one receptor. To overcome this, we initially used a UC cell line, 94-10, which expresses relatively low levels of FGFRs 2, 3 and 4 and almost undetectable levels of FGFR1. FGFR1 was ectopically expressed in a polyclonal population of 94-10 cells by retroviral transduction and expression confirmed by western blotting (Figure S1D)). Cells expressing FGFR1 (94-10-FR1) were stimulated with heparin or heparin and FGF2 for 72 h. 94-10-FR1 cells cultured with FGF2 developed an elongated and larger morphology and appeared scattered compared to control cells, suggesting that they had undergone an EMT (Figure 1A). 94-10 wild type cells showed no response to FGF2.

Many studies have characterised phenotypic changes that occur during the EMT process. These include re-organisation of the actin cytoskeleton, increased migration and invasion and decreased expression of E-cadherin. No changes in morphology, actin cytoskeleton or invasion were observed in 94-10 cells transduced with a control vector. However, 94-10-FR1 cells cultured with FGF2 showed an altered actin cytoskeleton after 72 h (Figure 1B). Re-organisation of the actin cytoskeleton occurred rapidly after FGFR1 stimulation, with increased formation of stress-fibres by 30 minutes (Figure S1A). Flow cytometry confirmed an increase in cell size (Figure S1B). Migration was assessed using transwell (Figure 1C) and scratch-wound assays (Figure S1C). The transwell assay demonstrated that 94-10-FR1 cells migrate towards FGF2. Invasion was measured by plating the cells on a bed of 50% matrigel containing heparin or heparin and FGF2 (Figure 1D). Cells were stained for actin and imaged using confocal microscopy. Under normal conditions 94-10 cells form spheroids. In the presence of FGF2, cells from 90% of spheroids expressing FGFR1 invaded in streams into the matrigel by 96 h. 94-10-FR1 cells cultured with heparin and FGF2 also showed decreased expression of E-cadherin mRNA and protein (Figure 1E and F).

**Figure 2:**
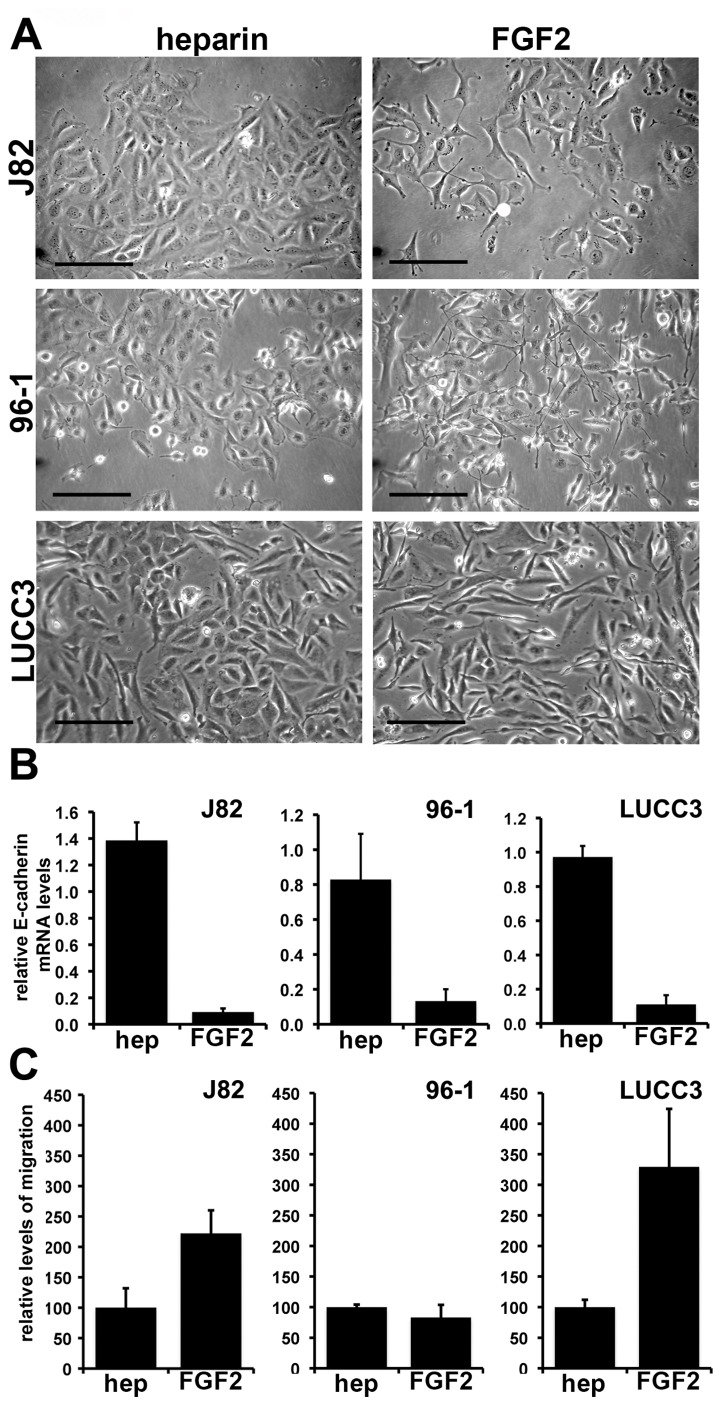
FGFR1 activation induces EMT in multiple UC cell lines. A. J82, 96-1, and LUCC3 expressing ectopic FGFR1 were cultured with heparin or heparin and FGF2. Images were taken at 72 h (Bars = 100 µm). B. Real time RT-PCR for E-cadherin on cell lines cultured with heparin or heparin and FGF2 for 72 h. Levels were normalised to SDHA and represented relative to untreated controls. C. Transwell assays were used to assess changes in migration. Migrated cells were stained with DAPI, imaged and total staining quantified using Volocity software. Values represent relative intensity of DAPI stain compared to the heparin only treated cells.

To determine the generality of FGFR1-induced EMT in UC, FGFR1 was expressed in five additional UC cell lines (J82, LUCC3, 96-1, 97-7 and VMCUB3). Ectopic expression of FGFR1 followed by FGF2 treatment induced an EMT in 3 of the 5 lines (J82, LUCC3, and 96-1) (Figure 2). These cell lines express higher endogenous levels of FGFR1 than 94-10 (96-1>J82>LUCC3) and treatment with FGF2 even in the absence of ectopic expression of FGFR1 resulted in an EMT-like phenotype (data not shown). Activation of FGFR1 caused altered morphology and cell scattering (Figure 2A), and a significant (Student’s T-test, p<0.05) decrease in E-cadherin mRNA levels in all three cell lines (Figure 2B). The effect of FGFR1 on cell migration was measured using transwell assays. FGFR1 activation significantly (p<0.05) increased the level of migration of J82 and LUCC3 but not 96-1 which were found to have a high basal rate of migration independent of FGF stimulation (Figure 2C).

### FGFR1 Activates Multiple Signalling Pathways in Urothelial Carcinoma Cells

**Figure 3:**
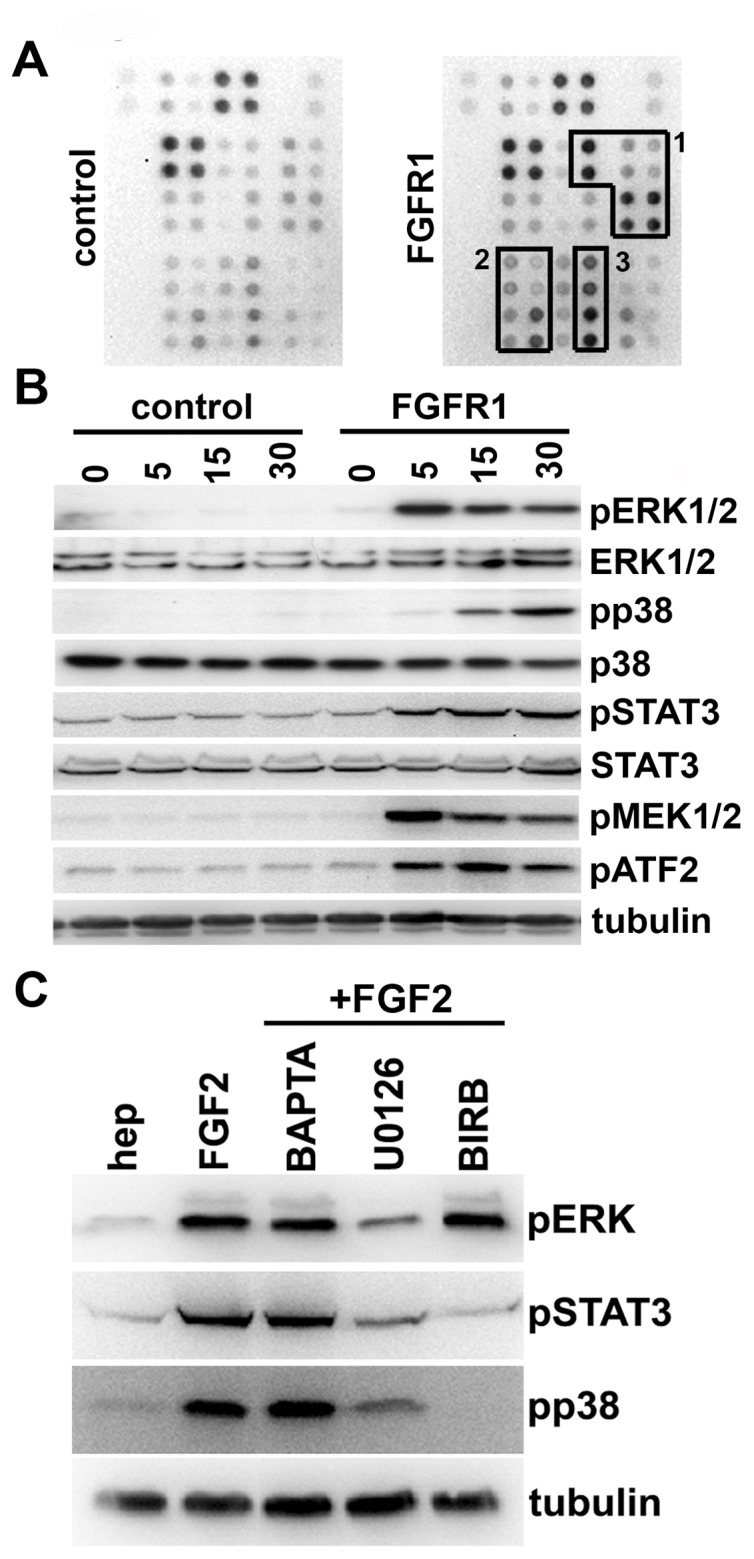
Signaling downstream of FGFR1 in 94-10 cells. A. The Human Phospho-Kinase Array was performed to examine FGFR1 signalling. Boxes 1, 2 and 3 represent activated proteins from the MAPK, STAT and downstream effectors of MAPK induced signaling cascades, respectively. B. Confirmation by Western blotting of the phosphorylated proteins identified in the Human Phospho-Kinase Array. Vector controls and FGFR1-expressing cells were cultured with heparin and FGF2 for indicated time periods (minutes). Lysates were harvested and used for Western blotting. C. Western blots showing inhibition of specific pathways using small molecule inhibitors. 94-10-FR1 cells were cultured with BAPTA-AM, U0126 or BIRB for 1 hr prior to addition of FGF2 for 10 min.

We used Human Phospho-Kinase Antibody Arrays to identify the signalling pathways activated by FGFR1 in 94-10-FR1 (Figure 3A). Cells were cultured with FGF2 for 30 minutes and extracts analysed on the array. ERK and p38 MAPKs (Figure 3A box 1) and STAT3/5/6 (Figure 3A box 2) signalling pathways were activated. Downstream effectors of these pathways were also activated (Figure 3A boxes 1 & 3). Western blotting confirmed activation of these pathways (Figure 3B). We have shown previously that the MAPK pathway is activated by FGFR1 in telomerase-immortalised normal human urothelial cells (TERT-NHUC), though in these cells an EMT is not induced [15]. Interestingly, little activation of STATs was observed on an array performed on normal human urothelial cells (NHUC) (data not shown).

Previous studies have demonstrated that increased activation of ERK, p38 and STATs is associated with induction of EMT [28]. We used a range of small molecule inhibitors to determine which pathways are important for FGFR1-induced urothelial EMT (Figure 3C). The MAPK pathway was inhibited using U0126, and this also prevented p38 and STAT3 phosphorylation. p38 phosphorylation was inhibited using BIRB, and this was associated with reduced STAT3 phosphorylation but no change in ERK phosphorylation. Our previous studies demonstrated that PLCγ plays an important role in FGFR-induced phenotypes in TERT-NHUC [21], [29]. PLCγ signalling activates the MAPK pathway but is also involved in regulating intracellular free calcium levels [30] and PLCγ-mediated mechanisms are known to strongly influence migration via regulation of the cytoskeleton [31]. To determine if PLCγ was regulating the cytoskeletal changes via modulation of calcium flux, we cultured cells with the calcium chelator BAPTA-AM and found no effects on signalling. Overall these results suggest that calcium flux modulation mediated by PLCγ may not regulate ERK, but that p38 and STAT3 are regulated downstream of ERK under these conditions Further work is required to exclude off-target effects of BIRB.

### PLCγ and ERK Co-operate to Regulate Different Aspects of Urothelial EMT

**Figure 4:**
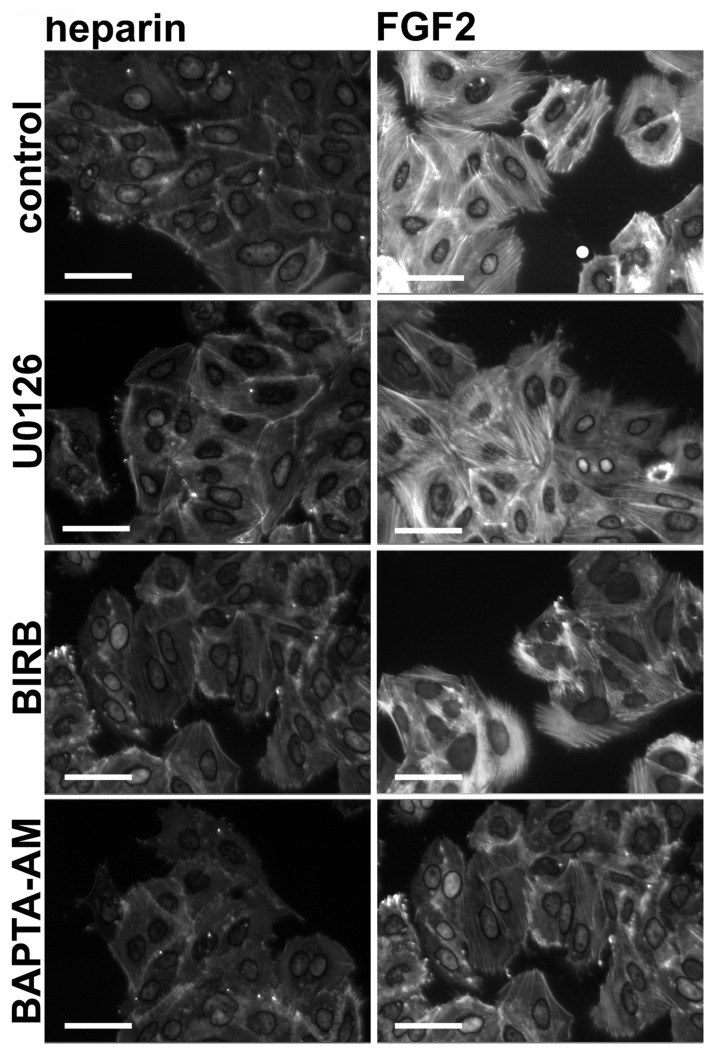
FGFR1-induced cytoskeletal changes are calcium-dependent. 94-10-FR1 cells were cultured with inhibitors for 1 h prior to addition of heparin or heparin and FGF2 for 2 h. Cells were fixed, stained with Phalloidin and DAPI, and imaged (bars = 30 µm). Images represent combined DAPI and Phalloidin staining.

An important change that occurs during EMT and is essential for increased cell migration is altered regulation of the actin cytoskeleton. 94-10-FR1 cells were pre-cultured with BAPTA-AM, U0126 or BIRB for 1 h and then treated with FGF2 for 2 h (Figure 4) to identify pathways responsible for regulating early changes in the actin cytoskeleton. Our results indicated that the MAPK and the p38/STAT3 pathway do not regulate actin cytoskeleton reorganisation in response to FGFR1 signalling. However treatment with BAPTA-AM completely abrogated stress fibre formation, indicating a possible role for calcium in this process. Further work to assess calcium fluxes caused by FGF2 is needed to confirm the role of calcium.

**Figure 5:**
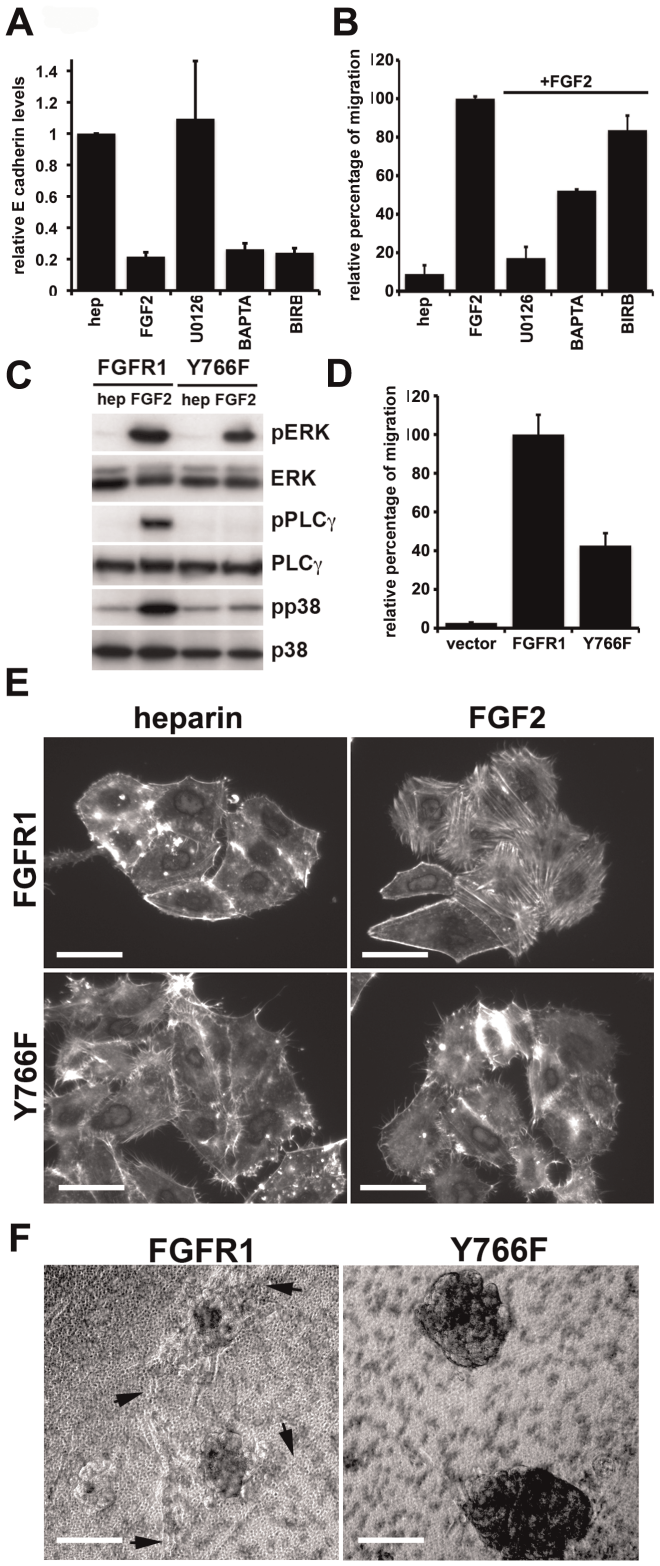
Regulation of E-cadherin and migration. A. Real time RT-PCR for E-cadherin. levels. 94-10-FR1 cells were cultured in the presence of heparin or heparin and FGF2 with or without U0126, BAPTA-AM or BIRB for 72 h. mRNA was harvested, cDNA made and used for real-time PCR. Levels were normalised to SDHA and standardised to heparin control. B. The effect of the inhibitors on migration was measured by transwell assay. Cells were seeded in the upper chamber, pretreated with inhibitors for 1 hr and fixed and stained after 24 h. Values represent percentage intensity of DAPI stained migrated cells compared to cells cultured with FGF2. C. Western blot showing activation of pathways in 94-10-FR1 and 94-10-Y766F expressing cells. Cells were cultured with either heparin or heparin and FGF2 for 10 min. D. Transwell assay for 94-10 vector control, 94-10-FR1 and 94-10-Y766F cells cultured with heparin and FGF2. Values represent percentage intensity of DAPI stain in the lower chamber compared to 94-10-FR1 cells. E. 94-10-FR1 and 94-10-Y766F cells were cultured with heparin or heparin and FGF2 for 2 h. Cells were stained with DAPI and Phalloidin to examine changes in FGF2-induced actin cytoskeleton (bars = 30 µm). F. FGFR1 and Y766F cells were grown on matrigel in a transwell for 96 h before imaging. FGF2 was used as a chemoattractant in the lower chamber. Arrows indicate regions of invasion (bars = 0.5 mm).

We then set out to identify which of these pathways effect changes in E-cadherin expression. 94-10-FR1 cells were cultured with BAPTA-AM, U0126 or BIRB and FGF2 for 72 h (Figure 5A). Chelating calcium or inhibiting p38/STAT3 activation did not prevent FGFR1-induced repression of E-cadherin levels. However, prevention of MEK1/2 activation completely abrogated repression of E-cadherin expression. This suggests that ERK activation is the main downstream effector of FGFR1 that regulates E-cadherin transcription.

Next we examined migration. Both U0126 and BAPTA-AM significantly (p<0.05) reduced migration through transwell membranes (Figure 5B). This suggests that both MAPK activation and the change in the actin cytoskeleton induced by PLCγ and/or calcium are important for FGF2-induced chemotaxis. To further validate PLCγ as an important regulator of migration and EMT, a mutant form of FGFR1, that does not contain a binding site for PLCγ (Y766F) [21], was expressed in 94-10 cells. 94-10-FR1 and 94-10-Y766F expressed equivalent levels of FGFR1 protein (Figure S2D), but 94-10-Y766F cells showed lower levels of ERK and p38 phosphorylation in response to FGF2 (Figure 5C). We observed a similar effect previously in TERT-NHUC [21]. We confirmed that activation of Y766F did not lead to phosphorylation of PLCγ (Figure 5C). 94-10-Y766F showed a significantly (p<0.05) reduced level of migration (Figure 5D) and no reorganisation of the actin cytoskeleton (Figure 5E), indicating that PLCγ activation is important for modulating migration via reorganisation of the actin cytoskeleton during FGFR1-induced EMT.

Furthermore, culture of 94-10-Y766F with FGF2 did not cause morphological and size changes (Figure S2A and B), E-cadherin levels were not reduced as much as in 94-10-FR1 cells (Figure S2C) and migration into Matrigel™ was reduced (Figure 5F).

### COX-2 Mediates FGFR1-induced Migration

**Figure 6:**
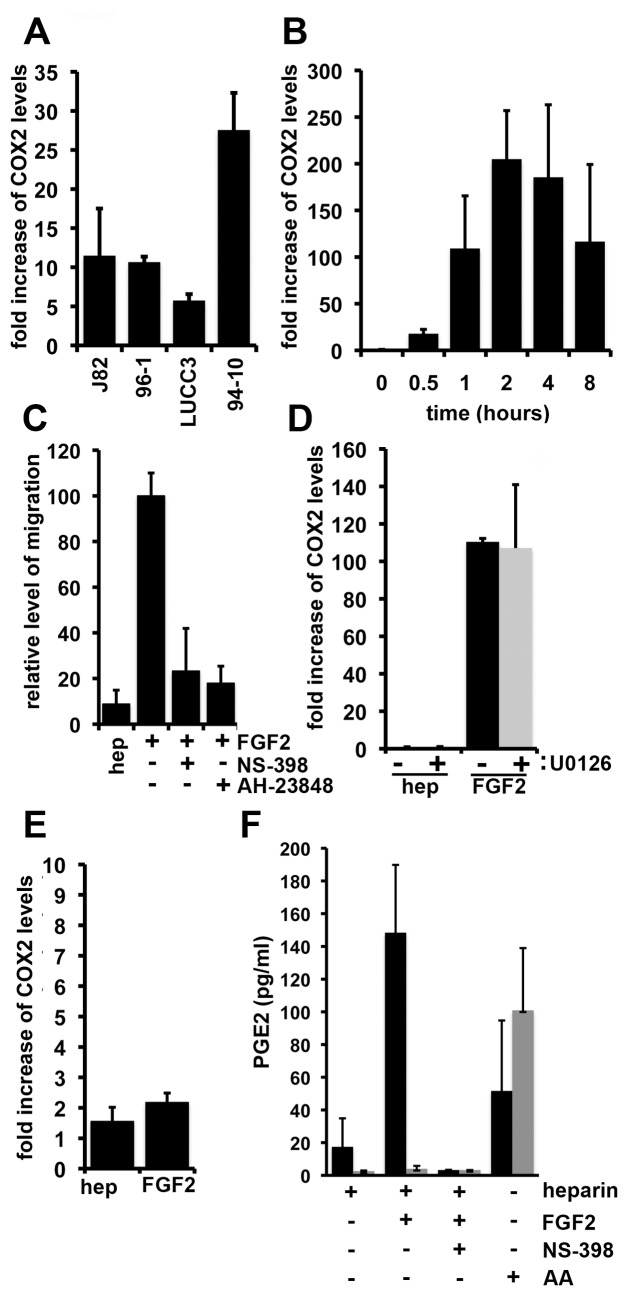
FGFR1 induces expression of COX-2. A. Real time RT-PCR for COX-2 on cell lines expressing ectopic FGFR1 cultured with heparin and FGF2 for 24 h. Values were standardised to cells cultured with heparin alone and represent fold increase compared to controls. B. Real-time RT-PCR for COX-2 in 94-10-FR1 cells cultured with heparin and FGF2 for the indicated time points. Results are represented as fold change compared to untreated cells. C. Transwell assays comparing the effects of COX-2 and EP4 inhibitors on FGFR1-induced migration. Values represent relative percentage of migrated cells (as measured by DAPI stain) compared to 94-10-FR1 cells cultured with heparin and FGF2. D. Real time RT-PCR for COX-2 in 94-10-FR1 cells cultured with heparin or heparin and FGF2 for 1 h. Cells were pre-treated for 1h with and without U0126 and results are represented as a fold-change compared to heparin alone. E. Real-time RT-PCR for COX-2 expression in 94-10-Y766F cells cultured with heparin and FGF2 for 1 h. All real time RT-PCR experiments were normalised to SDHA. F. PGE2 levels were measured by an enzyme immunoassay in lysates (black bars) and media (gray bars) from cells cultured with heparin; heparin and FGF2; heparin, FGF and NS-398; or arichidonic acid (AA) for 4 h.

EMT is a complex process that potentially involves a global change in gene expression. To identify genes regulated by FGFR1, we used microarray analysis to compare gene expression profiles of 94-10-FR1 cultured with and without FGF2 for 24 h. A literature search was performed to identify genes that were regulated by FGFR1 in this analysis that are also known to be associated with invasion in UC. Cyclooxygenase-2 (COX-2) expression was strongly induced by FGFR1 (46 fold increase, top 2% of increased genes). We confirmed this upregulation by real-time RT-PCR. Levels were also increased in J82, LUCC3, and 96-1 cell lines expressing ectopic FGFR1 (Figure 6A). Numerous previous reports have demonstrated an association of COX-2 with UC invasion [32], [33], [34], [35], [36]. We observed a 2.5-fold increase in expression of ZEB1, a recognised “driver” of EMT but no significant change in levels of other commonly reported markers of EMT (N-cadherin, vimentin and fibronectin).This might reflect the time at which the mRNA was harvested and further extended time courses are required to further elucidate changes in gene expression.

94-10-FR1 cells were used to examine the regulation of COX-2 by FGFR1 in more detail. FGFR1 activation led to an increase in COX-2 mRNA of 205-fold by 2 h (Figure 6B). Transcriptional, posttranscriptional and posttranslational mechanisms are known to regulate COX-2 expression [37]. Further work is need to study the precise mechanisms involved in FGFR1-induced activation of COX-2.

Next we used NS-398, a selective inhibitor of COX-2, to determine whether increased COX-2 expression contributed to FGFR1-induced migration (Figure 6C). NS-398 significantly (p<0.05) reduced the level of migration and interestingly prevented FGFR1-induced repression of E cadherin expression increasing the level of E cadherin above control levels (data not shown). A similar decrease in migration was observed in cells pre-treated with AH-23848, an inhibitor of the PGE2-EP4 receptor, further demonstrating that prostaglandin synthesis is important in this process. Repression of E-cadherin expression was also prevented (data not shown). We also overexpressed COX-2 in 94-10 cells and cultured cells with PGE2 but this did not promote migration or EMT (data not shown). After further examination of the microarray we observed that FGFR1 activation also increased EP4 and phospholipase A2 (PLA2) expression levels. EP4 is a receptor for prostaglandins and PLA2 is involved in releasing arachindonic acid, which is a precursor in the production of prostaglandins by COX-2.

As we had shown that multiple signalling pathways co-operate to promote EMT, we examined which of these was responsible for COX-2 regulation. Inhibition of the MAPK pathway did not prevent the increase in COX-2 expression (Figure 6D). However, inhibition of PLCγ activation using the Y766F mutant prevented increased COX-2 expression (Figure 6E), demonstrating that PLCγ regulates FGFR1-induced COX-2. We then measured the levels of intracellular and secreted PGE_2_ in 94-10 FR1 cells cultured with FGF2. As a control for PGE2 synthesis we also cultured 94-10 FR1 cells with arachidonic acid (AA). AA caused an increase in intracellular and secreted PGE_2_ levels. FGFR1 stimulated a significant (p<0.05) increase in intracellular but not secreted PGE_2_ levels and this was dependent on COX-2 activity, as shown by the prevention of PGE_2_ synthesis by NS-398 (Figure 6F).

## Discussion

Recent publications have highlighted important roles for FGF receptors in UC. The majority of studies have focused on FGFR3 but our recent findings also implicate FGFR1 [21]. It is clear that there are at least two major groups of bladder tumours that develop via distinct molecular pathways: low-grade non-invasive (stage Ta) and invasive (≥ stage T2) tumours [16], [17]. Increased expression of FGFR1 is found in both of these groups [15]. Previously we showed that upregulated FGFR1 signalling promotes proliferation and survival of normal human urothelial cells, suggesting that FGFR1 could contribute to hyperplasia early in the development of low-grade non-invasive UC [21]. Some growth factors, including TGFβ and HGF, are known to regulate proliferation or differentiation under normal conditions but also induce EMT-specific events in pre-malignant or malignant epithelial cells [9]. This raised the question of whether FGFR1 signalling may play different roles in the two distinct UC sub-groups.

Our findings indicate that this is the case. Expression of FGFR1 in a UC cell line that expresses relatively low levels of FGFRs induced EMT, indicating a context-dependent effect of FGFR1 in urothelial tumor cells. In three of five other tumor cell lines tested, this was also the case. Even without expression of ectopic FGFR1, these cells could undergo FGF2-induced EMT, suggesting that if FGF was present in the tissue microenvironment *in vivo*, the tumours from which these cell lines were established may have expressed an FGFR-dependent EMT phenotype. The two cell lines that did not undergo EMT expressed higher levels of E-cadherin (data not shown), raising the possibility that for FGFR1 to induce EMT, other events that confer a partially de-differentiated state may be required. Also, our unpublished data shows there is an inverse correlation between FGFR1 and E-cadherin expression in our cell line panel demonstrating a potential link between FGFR1 and EMT. In this context, it is interesting to note that PPARγ, a known regulator of urothelial differentiation *in vitro* [38], is implicated as a negative feedback regulator of COX-2 [39], [40].

FGF signalling is known to regulate EMT during development and in models of cancer. FGFs play an integral part in regulating migration and patterning of mesoderm during development and FGFR1 orchestrates the EMT of the mesoderm at the primitive streak by regulating E-cadherin expression [41]. FGFR1 activation promotes EMT in rodent models of breast [42] and prostate [22] cancer. Our data further broadens the range epithelial cancers in which FGFR1 is implicated in EMT.

Other studies on bladder carcinoma cell lines have implicated FGFs in regulation of EMT. A rat bladder carcinoma cell line, NBT-II, can undergo EMT following addition of several growth factors including FGF1, FGF7, and FGF10 [43] and NBT-II carcinoma cell lines that were rendered autocrine for FGF1 activation had a much higher level of tumorigenicity [44]. Interestingly these cells showed a dual response to FGF1, with promotion of proliferation or induction of EMT depending on degree of confluence and cyclic AMP (cAMP) levels [45]. At low density and low cAMP levels FGF1 induced EMT but at confluence and high cAMP levels FGF1 promoted proliferation. Whether cAMP levels influence FGFR1-induced phenotypes in human bladder cancer cell lines needs to be investigated.

Our results indicate that FGFR1 activates several signalling pathways including activation of ERK, p38 and STATs. Previous studies of these pathways have demonstrated their importance in promoting EMT [46], [47], [48]. To identify the pathway(s) responsible for inducing EMT we used a range of inhibitors to determine their effect on FGFR1-induced phenotypic and functional changes. MEK1/2 inhibition by U0126 prevented FGFR1-induced EMT, reducing both FGFR1-driven migration and regulation of E-cadherin, strongly suggesting that FGFR1 exerts its effects on cell morphology and oncogenic functions via regulation of the MEK-ERK pathway. Similarly, the RAS-MEK-ERK cascade appears to be mediate TGFβ-induced EMT in pancreatic cancer cells [49].

We found that PLCγ activation also plays an integral part in regulating FGFR1-induced urothelial EMT. Activation of PLCγ regulated remodelling of the actin cytoskeleton that effected morphological change and promoted migration. Modulation of PLCγ effects using the calcium chelator BAPTA-AM or by expressing a mutant form of FGFR1 that cannot activate PLCγ [50] prevented actin cytoskeleton remodelling and reduced migration. PLCγ is highly expressed in several tumour types [51], [52] and has been shown to play a critical role in cell migration and invasion [53], [54], [55]. One study has shown that PLCγ activation is not required for FGF-induced motility in L6 and CHO cell lines [56]. Thus, these non-cancer derived cell lines respond similarly to normal human urothelial cells in which we have shown no effect of PLCγ activation on migration (data not shown). This implies that a molecular switch occurs during cancer development that facilitates the effect of PLCγ on migration and invasion.

We found that COX-2 was strongly upregulated following FGFR1 activation. Immunohistochemistry analyses have demonstrated that COX-2 expression is increased in invasive UC [32], [34], [35], [36] and that the highest levels of expression are found observed at the invasive front [33], suggesting COX-2 is intimately involved in regulating UC invasion. A recent paper also describes a reciprocal correlation between expression of COX-2 and E-cadherin in UC [57]. Our results add weight to these observations and identify a mechanism by which COX-2 may be regulated in the significant proportion of aggressive UC that express high levels of FGFR1 [21].

Activated FGFR1 increased intracellular PGE2 but we could not detect an increase in secreted PGE2. However, culture with arachidonic acid, showed that these cells can metabolise this precursor to produce secreted PGE2. This may indicate the existence of a novel FGF-dependent COX-2 signalling pathway that causes an increase in intracellular PGE2 levels that is involved in regulating migration.

Our results indicate that PLCγ mediates both upregulation of COX-2 and downregulation of E-cadherin, but to a lesser extent than the MAPK pathway. Inhibition of COX-2 using NS-398 in this system prevented FGFR1-induced repression of E-cadherin. Jang *et al* have shown a similar effect in a bladder cancer cell line, with ectopic expression of COX-2 or culture with PGE2 leading to reduced E-cadherin expression [57]. Cells lacking PLA2 show lower levels of arachidonic acid, and cytosolic calcium may also have a regulatory effect on PLA2 activity [58]. As PLCγ is involved in regulating calcium flux and the calcium chelator BAPTA-AM repressed migration, this suggests FGFR1 coordinates activation of multiple proteins and molecules involved in prostaglandin synthesis which is an important pathway that mediates migration and EMT.

In conclusion our data strongly suggests that FGFR1 regulates different processes in the two types of UC. As previously described FGFR1 expression in non-invasive UC promotes proliferation and survival [21], our data now demonstrates that in invasive UC FGFR1 mediates EMT and invasion. By deciphering the mechanisms that promote this phenotype our study has identified that FGFR1 activation of the MAPK pathway is critical for EMT but PLCγ activation also contributes to the migratory phenotype. To our knowledge this is the first paper to describe that FGFR1 activation promotes prostaglandin synthesis in UC. We have also identified numerous potential therapeutic targets (FGFR1, MAPK, PLCγ and COX-2). We envisage inhibition of FGFR1 could benefit patients suffering from both non-invasive and invasive subtypes while COX-2 inhibition could benefit patients diagnosed with invasive disease. A clinical trial utilising COX-2 inhibitor is in progress and it will be interesting to see the clinical benefit of this inhibition.

## Supporting Information

Figure S1 A. Actin cytoskeletal changes in 94-10-FR1 cells cultured with FGF2 and fixed at the specific time points (minutes) shown (bars = 30 µm). B. Flow cytometry results indicating increased cell size (FSC) in FGF2 treated 94-10-FR1 cells. C. Scratch assay demonstrating increase migration of 94-10-FR1 cells compared to control (bars = 100 µm). D. Western blot showing expression of FGFR1 in 94-10 vector controls and cells expressing FGFR1.
(TIF)

Figure S2 A. 94-10-Y766F show little change in morphology after culture with FGF2 compared to 94-10-FR1 cells (bars = 100 µm). B. Flow cytometry showing no change in cell size of 94-10-Y766F cells culture with FGF2. C. Western blot showing E-cadherin and plakoglobin expression levels in 94-10-FR1 and 94-10-Y766F cells cultured with heparin or heparin and FGF2 for 72 h. Tubulin was used as loading control. D. Western blot showing levels of FGFR1 protein in 94-10-FR1 and 94-10-Y766F cells. Tubulin was used as loading control.
(TIF)
