# Supplementary figures and images for: FGFR1-Induced Epithelial to Mesenchymal Transition through MAPK/PLCγ/COX-2-Mediated Mechanisms
Source: PLoS One. 2012 Jun 12;7(6):e38972. doi: 10.1371/journal.pone.0038972 (PMC3373505; doi:10.1371/journal.pone.0038972)

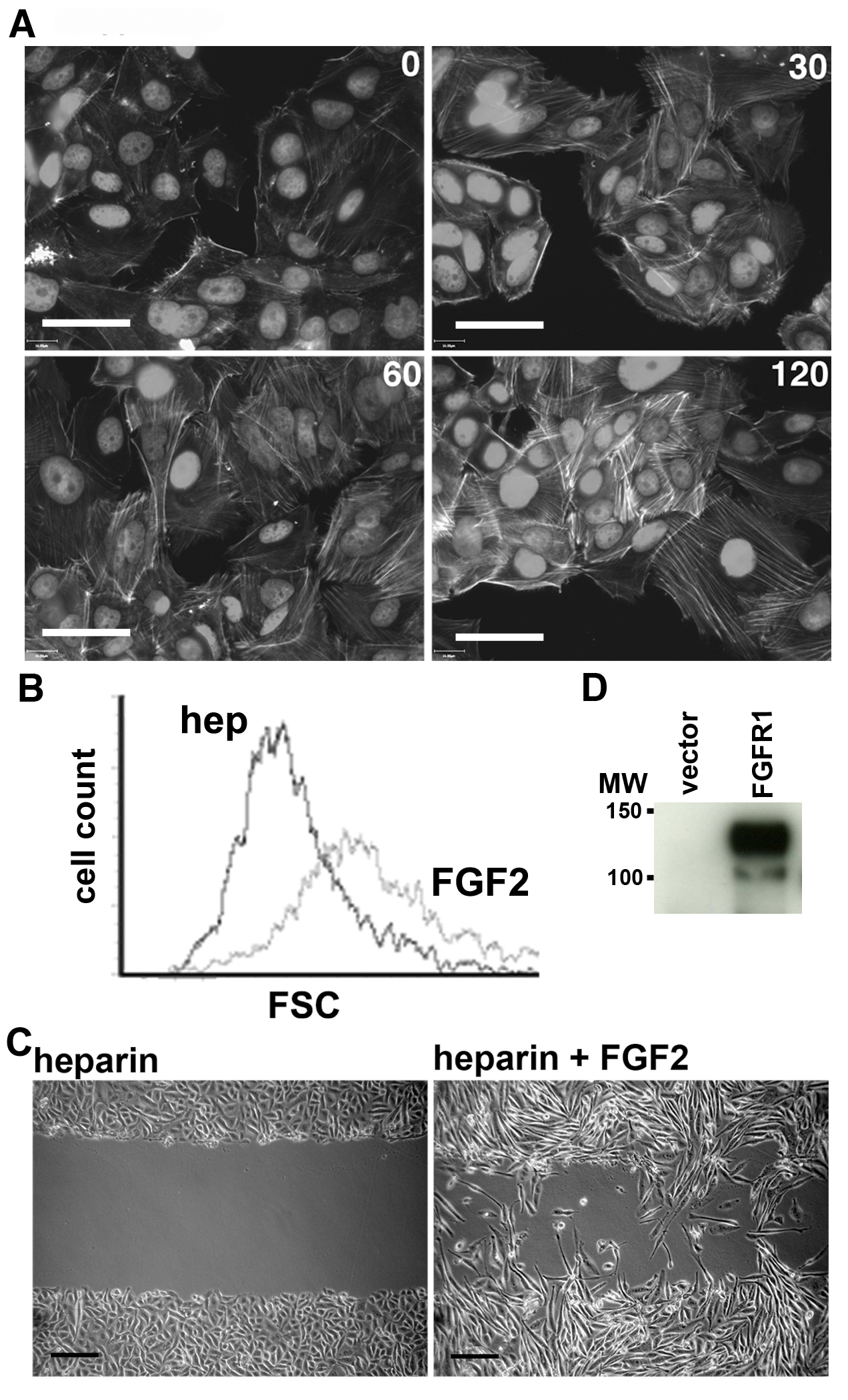

Supplement: Figure S1 — A. Actin cytoskeletal changes in 94-10-FR1 cells cultured with FGF2 and fixed at the specific time points (minutes) shown (bars = 30 µm). B. Flow cytometry results indicating increased cell size (FSC) in FGF2 treated 94-10-FR1 cells. C. Scratch assay demonstrating increase migration of 94-10-FR1 cells compared to control (bars = 100 µm). D. Western blot showing expression of FGFR1 in 94-10 vector controls and cells expressing FGFR1. (TIF) [file pone.0038972.s001.tif]

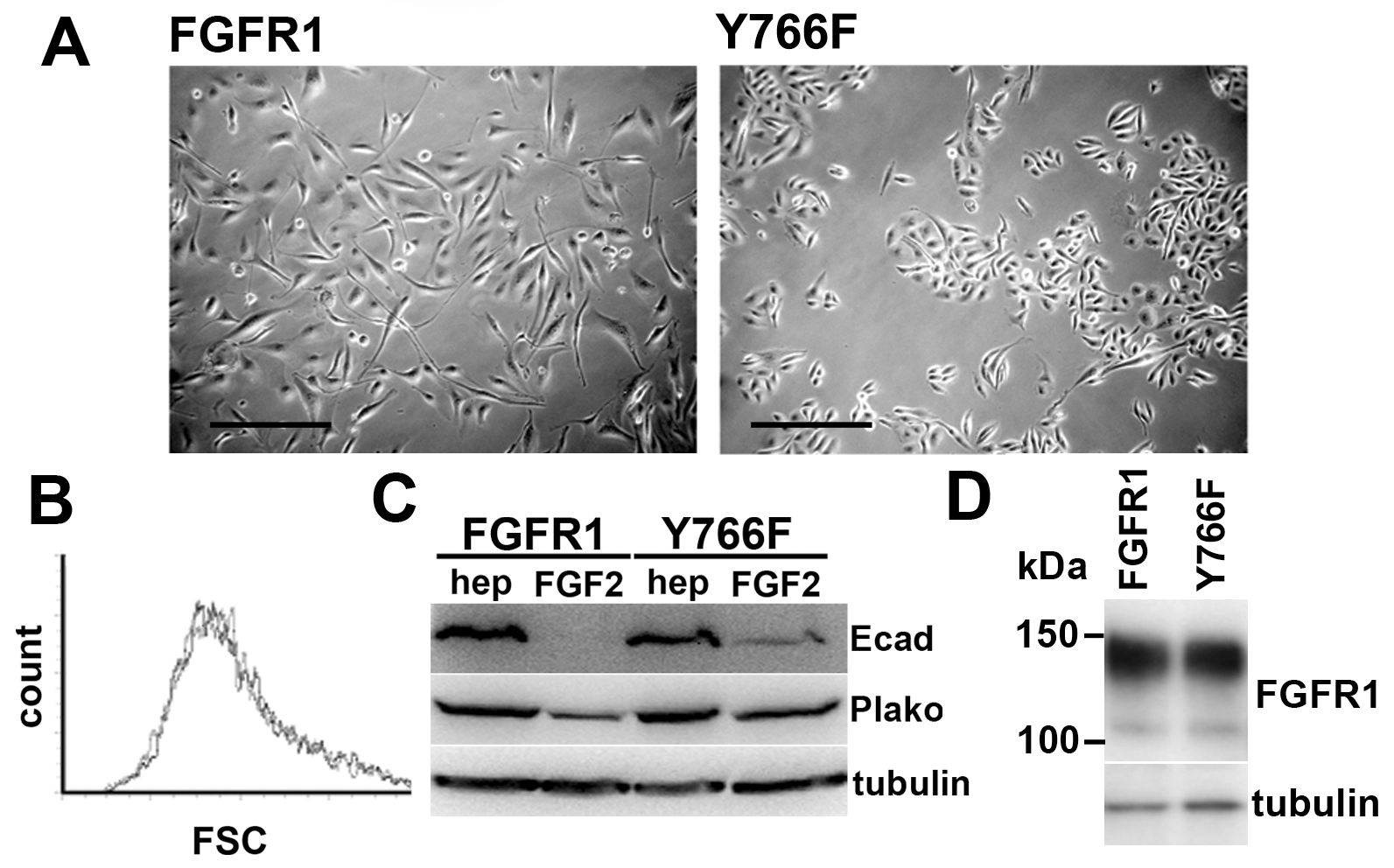

Supplement: Figure S2 — A. 94-10-Y766F show little change in morphology after culture with FGF2 compared to 94-10-FR1 cells (bars = 100 µm). B. Flow cytometry showing no change in cell size of 94-10-Y766F cells culture with FGF2. C. Western blot showing E-cadherin and plakoglobin expression levels in 94-10-FR1 and 94-10-Y766F cells cultured with heparin or heparin and FGF2 for 72 h. Tubulin was used as loading control. D. Western blot showing levels of FGFR1 protein in 94-10-FR1 and 94-10-Y766F cells. Tubulin was used as loading control. (TIF) [file pone.0038972.s002.tif]
